# Mild Hyperthermia Aggravates Glucose Metabolic Consequences in Repetitive Concussion

**DOI:** 10.3390/ijms21020609

**Published:** 2020-01-17

**Authors:** Meghan Blaya, Jessie Truettner, Weizhao Zhao, Helen Bramlett, William Dalton Dietrich

**Affiliations:** 1 The Miami Project to Cure Paralysis, Department of Neurological Surgery, University of Miami Miller School of Medicine, Miami, FL 33136, USA; moconnell2@med.miami.edu (M.B.); jtruettner@med.miami.edu (J.T.); hbramlett@miami.edu (H.B.); 2 Department of Biomedical Engineering, University of Miami Miller School of Medicine, Miami, FL 33146, USA; wzhao@med.miami.edu; 3 Bruce W. Carter Department of Veterans Affairs Medical Center, Miami, FL 33136, USA

**Keywords:** traumatic brain injury, mild TBI, concussion, repetitive concussion, 2DG, hyperthermia, glucose metabolism

## Abstract

Traumatic brain injury (TBI) is one of the leading causes of mortality and disability around the world. Mild TBI (mTBI) makes up approximately 80% of reported cases and often results in transient psychological abnormalities and cognitive disruption. At-risk populations for mTBI include athletes and other active individuals who may sustain repetitive concussive injury during periods of exercise and exertion when core temperatures are elevated. Previous studies have emphasized the impact that increased brain temperature has on adverse neurological outcomes. A lack of diagnostic tools to assess concussive mTBI limits the ability to effectively identify the post-concussive period during which the brain is uniquely susceptible to damage upon sustaining additional injury. Studies have suggested that a temporal window of increased vulnerability that exists corresponds to a period of injury-induced depression of cerebral glucose metabolism. In the current study, we sought to evaluate the relationship between repetitive concussion, local cerebral glucose metabolism, and brain temperature using the Marmarou weight drop model to generate mTBI. Animals were injured three consecutive times over a period of 7 days while exposed to either normothermic or hyperthermic temperatures for 15 min prior to and 1 h post each injury. A ^14^C-2-deoxy-d-glucose (2DG) autoradiography was used to measure local cerebral metabolic rate of glucose (lCMR_Glc_) in 10 diverse brain regions across nine bregma levels 8 days after the initial insult. We found that repetitive mTBI significantly decreased glucose utilization bilaterally in several cortical areas, such as the cingulate, visual, motor, and retrosplenial cortices, as well as in subcortical areas, including the caudate putamen and striatum, compared to sham control animals. lCMR_Glc_ was significant in both normothermic and hyperthermic repetitive mTBI animals relative to the sham group, but to a greater degree when exposed to hyperthermic conditions. Taken together, we report significant injury-induced glucose hypometabolism after repetitive concussion in the brain, and additionally highlight the importance of temperature management in the acute period after brain injury.

## 1. Introduction

Traumatic brain injury (TBI), once referred to as the “silent epidemic”, is now well recognized as a critical public health and socioeconomic problem. In 2013, approximately 2.8 million TBI-related emergency department visitations, hospitalizations, and deaths occurred in the United States [1]. However, this number likely underestimates the true number of TBIs due to individuals with mild TBI (mTBI) not seeking medical care or being treated in outpatient or non-civilian facilities [2]. TBI is defined as a biomechanically-induced disruption of neurological function within the cranial vault. mTBI makes up approximately 80% of reported cases and is commonly referred to as concussion [3]. Concussion is considered a subset of mTBI. However, the terms are often used interchangeably in the literature [4]. With mTBI, visualization of brain tissue appears normal and neuropsychological sequelae are often transient. However, clinical studies have shown that 10–40% of patients will go on to develop post-concussion syndrome [5,6]. Clinical complaints after mTBI can include headache, photophobia, amnesia, confusion, disorientation, dizziness, and vertigo—all of which may result with or without a loss of consciousness [7]. Some patients may acquire neuropsychiatric comorbidities, such as posttraumatic stress disorder, in the chronic period after the insult [8].

After mTBI, sequelae manifest due in part to biomechanical damage and injury-induced alterations in ionic and neurochemical cascades resulting in pathophysiological cellular and molecular changes. After traumatic insult, there is an initiation of unchecked ionic fluxes leading to overactivation of the Na^+^/K^+^ pump to restore ionic homeostasis [7]. In an attempt to meet the energy demand, cerebral metabolism is initially increased, leading to an upsurge in the local cerebral metabolic rate of glucose (lCMR_Glc_) in the acute hours after TBI. Glucose processing via the glycolytic pathway is the principle energy source of the brain. Disruption of these tightly regulated cellular processes disproportionately depletes ATP and contributes to worsened outcomes. Because these metabolic events may occur when there is an injury-induced decrease of cerebral blood flow, the resulting disparity between supply and demand can lead to regional states of “cellular energy crises” [7]. The acute increase in cerebral glucose utilization is subsequently followed by a metabolic depression lasting up to 10 days post mTBI in rats and up to 2–4 weeks in human [7]. These injury-induced hypometabolic changes in lCMR_Glc_ are widespread and coupled with cognitive dysfunction and spatial learning and memory deficits in both patients and preclinical animal models [7,9,10,11].

Athletes and military personnel are some of the most at-risk groups for brain injury [8,12]. In 2011, there were approximately 60,000 service members from the Army, Navy, Marine Corps, Air Force, and National Guard seeking clinical care for possible TBI-related conditions [13]. Coronado and colleagues [14] reported upwards of 450,000 sports- and recreation-related TBI emergency department visitations. However, because it has proven challenging to diagnose a mild brain injury using conventional assessment tools, athletes and military personnel are often cleared to return to normal activity without receiving appropriate medical care or consultation. The chances of sustaining a second or third concussion shortly thereafter are alarmingly high. Guskiewicz and colleagues [12] showed that collegiate football players with a history of concussion are 3.4 times more likely to sustain a second concussion within the same season. This potential for a second impact is important because several studies have shown that sustaining an additional injury when the brain is particularly susceptible can significantly exacerbate physiological damage and functional deficits [4,10,15,16,17]. Giza and DiFiori [4] reported that if the brain is injured during this “window”, there exists a cumulative effect of injurious processes and worsened neurological outcomes; however, when there is a greater interval between the mTBIs, cumulative deficits are not observed [18]. Importantly, this window of vulnerability coincides when glucose metabolism is depressed below baseline levels. Sustaining a TBI during injury-induced glucose hypometabolism can decrease lCMR_Glc_ to even lower values, exacerbating cellular damage, and leading to a slower recovery of post-concussive symptoms [10,12,19]. Thus, imaging techniques that reveal glucose metabolic changes may serve as important detectors to gauge the temporal window of susceptibility and elucidate when after mTBI it is safe to “return to play”.

In addition to the increased risk of repetitive head injury, another danger of mTBI in high-risk populations such as military service members and athletes lies in the possibility of increased central nervous system (CNS) temperature at the time of injury due to high physical activity levels. Furthermore, reactive pyrexia is a common feature after TBI. Thompson et al. [20] reported that 79% of TBI patients had at least one recorded fever while in the intensive care unit (ICU). The presence of fever in the acute phase after TBI has been associated with neurological severity, significantly worsened functional outcomes, increased intracranial pressure, lower Glasgow Coma Scores, and longer stays in the ICU [21,22]. Preclinical studies have also shown a similar relationship between acute elevated brain temperature and aggravated outcomes [23,24,25,26]. Truettner and colleagues [23] reported that elevated brain temperature after mTBI altered the polarization of resting, anti-inflammatory M2-like macrophages/activated microglia towards a more proinflammatory, hyperactive M1-like phenotype, which is associated with aggravation of neurological deficits and sustained functional consequences. Sakurai et al. [25] demonstrated that neuropathological damage was exacerbated when the brain was hyperthermic (39 °C) directly prior to mTBI (mimicking environmental influences that cause elevated temperature pre-injury) versus hyperthermic conditions after mTBI (mimicking posttraumatic pyrexia). Titus and colleagues [24] showed significant long-term cognitive deficits after mTBI under pre- and post-TBI hyperthermic conditions. Thus, in at-risk populations, hot environments and/or heat-retaining outerwear coupled with exertion or exercise, conditions that military service personnel and athletes are often exposed to, is of significant concern and an important area of study.

Hyperthermia-induced consequences after TBI are multifactorial and can include aggravated blood-brain barrier disruption, robust inflammatory responses, elevated cytokine release, enhanced generation of reactive oxidative species, as well as increased prevalence of excitotoxic events [26,27,28]. These consequences are attributed to a greater degree of contusion formation, diffuse axonal injury, tissue atrophy, susceptibility of cortical and hippocampal neurons to cell death, and, important to the scope of this study, glucose metabolic perturbations [25,28,29,30].

Because there appears to be a lack of knowledge in the field of glucose metabolism with regard to multiple, successive mTBI occurring while brain temperature is elevated, and because this is a commonly occurring phenomenon in certain high-risk populations, we sought to explore this relationship. In the present study, we assessed the metabolic state of the repetitively injured brain with and without exposure to hyperthermia.

## 2. Results

### 2.1. Physiology

Physiological variables were consistent throughout the duration of all three TBI surgeries and the ^14^C-2-deoxy-d-glucose (2DG) surgery. At time of trauma, repetitive mTBI normothermic animals were maintained between 36.7 and 36.9 °C while hyperthermic animals were kept between 38.6 and 38.9 °C (Table 1). There were no changes in respiratory status during induction of hyperthermia prior to TBI.

### 2.2. Qualitative Metabolic Consequences

**Figure 1:**
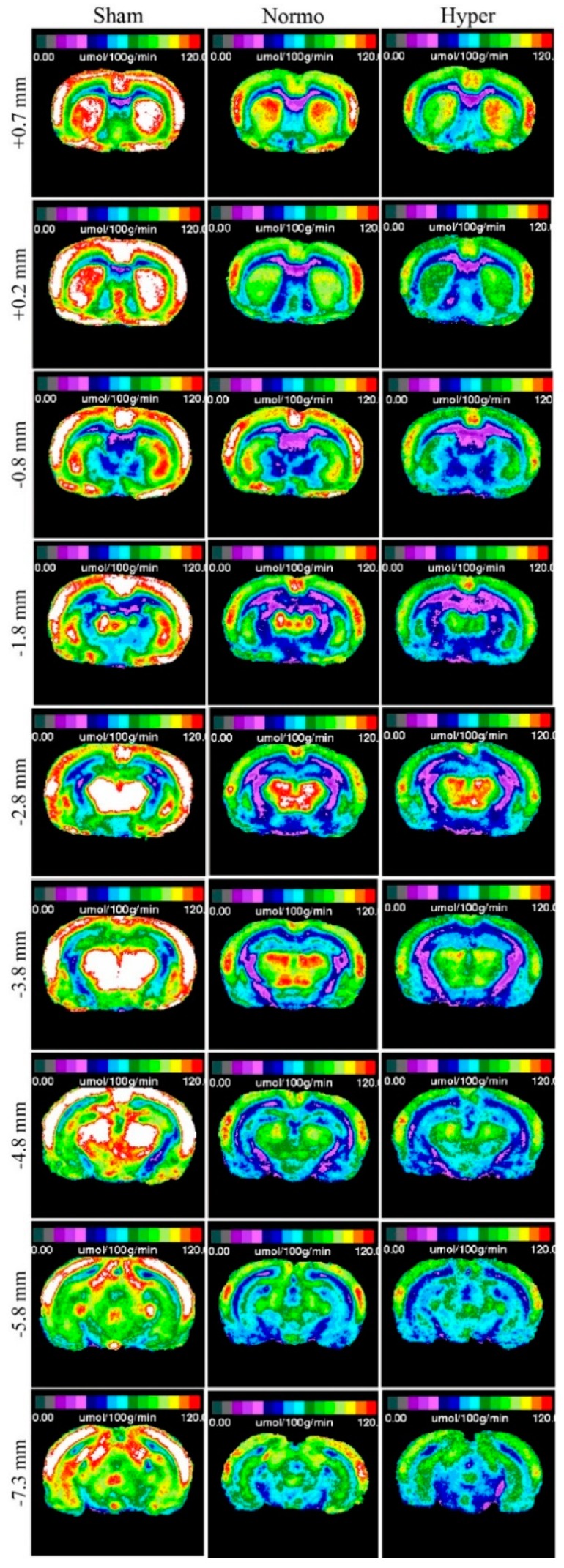
Quantitative autoradiographic images of local cerebral metabolic rate of glucose (lCMR_Glc_) displayed in pseudocolor. Each coronal level (with reference to bregma) is the average of *n* = 7 or 8 brains from the three experimental groups: sham (*n* = 7), normothermia traumatic brain injury (TBI; *n* = 8), and hyperthermia TBI (*n* = 8). After repetitive mild concussive TBI (3× over 7 days), there was a bilateral decrease in glucose utilization in both normothermic and, to a greater extent, hyperthermic animals in all nine bregma levels analyzed. Left hemisphere is shown on the left.

**Figure 2:**
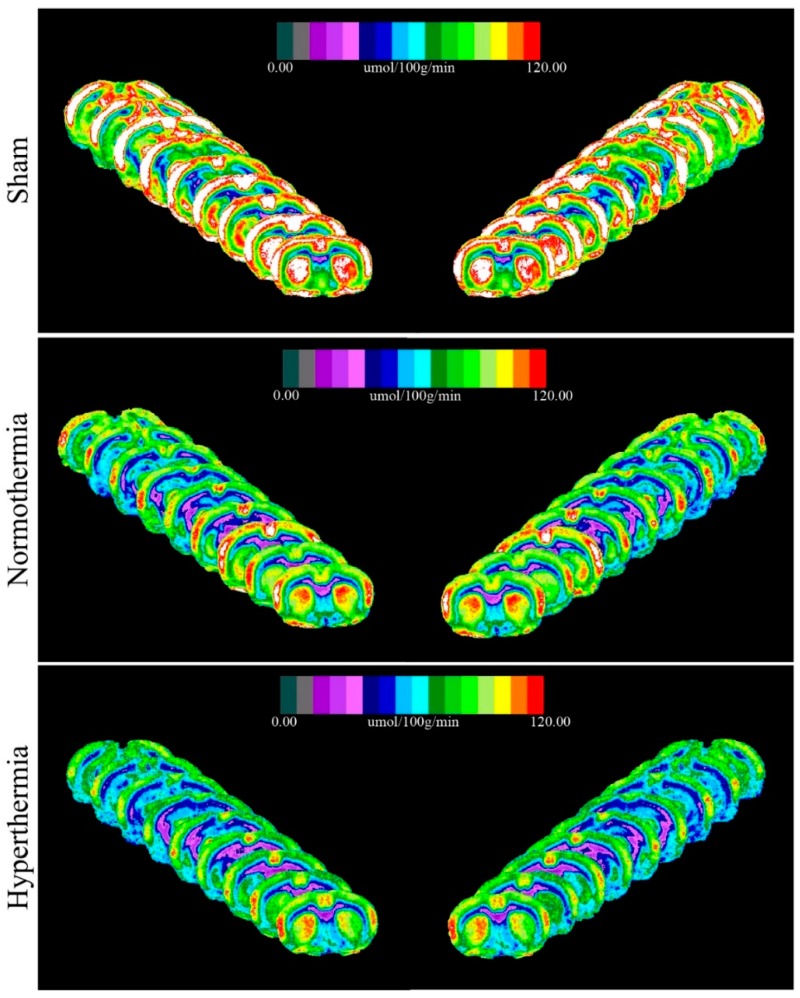
Stacked images of all nine bregma levels in mirrored orientations to show qualitative intergroup differences.

The closed-head weight drop model utilized in this study generates a mild diffuse concussive brain injury in the absence of focal brain lesions, and, thus, a clinically relevant tool to study repetitive concussion [14]. When assessing lCMR_Glc_ in this model, it is important to evaluate multiple bregma levels encompassing a large array of cortical and subcortical structures. We evaluated nine bregma levels encompassing 10 diverse regions of interest (ROIs). Autoradiographic images showed normal patterns of glucose utilization in sham animals (Figure 1, Left). lCMR_Glc_ levels were symmetrical in cortical and subcortical regions, typical of the awake rat in a resting-state. Three mild concussions over the course of one week (Day 0, Day 3, and Day 7 post initial injury) under normothermic conditions produced patterns of discernable depression in lCMR_Glc_ relative to sham control animals. In all nine bregma levels evaluated, glucose utilization was considerably diminished in both cortical and subcortical structures bilaterally (Figure 1, Center). We found that hyperthermia exposure at each mTBI showed an even greater degree of glucose hypometabolism with all nine bregma levels exhibiting this pattern (Figure 1, Right). In order to facilitate qualitative comparisons of lCMR_Glc_ intensity among groups, pseudocolor images were stacked in mirrored orientations (Figure 2).

### 2.3. Quantitative Findings

Two-way ANOVA and ROI pixel-based statistics confirmed visual observations that triple-hit repetitive mTBI significantly reduced glucose utilization relative to sham controls 8 days post initial TBI. When coupled with hyperthermia, there was an even greater degree of significant lCMR_Glc_ depression. We detected pronounced glucose utilization deficits between sham and repetitive mTBI normothermic and hyperthermia groups in seven or eight (respectively) of the nine bregma levels analyzed (Table 2).

**Figure 3:**
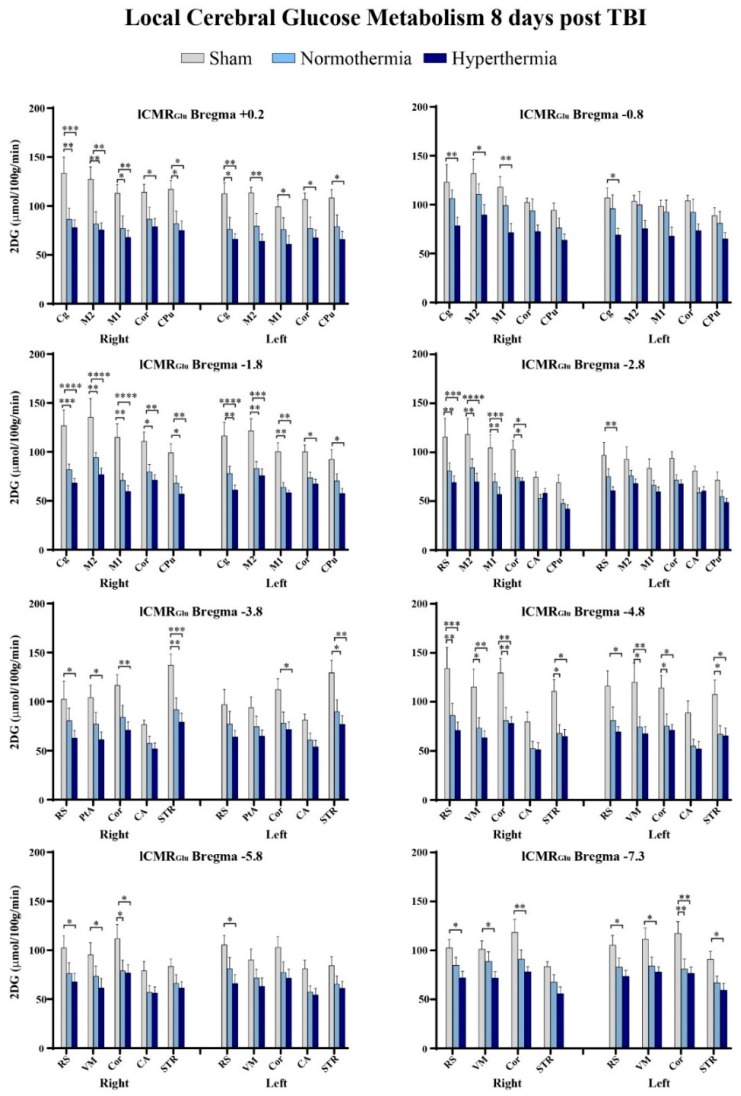
Eight out of nine bregma levels showed significantly decreased ^14^C-2-deoxy-d-glucose (2DG) values bilaterally in normothermic and hyperthermic repetitive mTBI animals relative to uninjured sham control animals. * *p* < 0.05, ** *p* < 0.01, *** *p* < 0.001, and **** *p* < 0.0001. CA: cornu ammonis/hippocampus, Cg: cingulate cortex, Cor: cortical strip, CPu: caudate putamen, M1: primary motor cortex, M2: secondary motor cortex), PtA: parietal association cortex, RS: retrosplenial cortex, STR: striatum, and VM: visual cortex.

Intergroup differences were further visualized using summary plots of the 2DG radiographic levels of various structures at select bregma levels (Figure 3). After repetitive mTBI, regardless of temperature group, we observed lCMR_Glc_ depression across all ROIs at all bregma levels. We found that certain bregma levels and ROIs were more significantly affected by repetitive injury than others. The cingulate cortex, retrosplenial cortex, primary and secondary motor cortices, as well as the cortical strip were some of these regions. The retrosplenial cortex in particular exhibited susceptibility to hyperthermia. Our quantitative data also showed that bregma level −1.8 encompassed the greatest number of ROIs that were significantly hypometabolic in glucose utilization relative to sham control animals. In the right cingulate cortex at −1.8 mm to bregma, there was a 36% decrease in 2DG levels in the repetitive mTBI normothermic group compared to sham animals, while hyperthermia reduced lCMR_Glc_ by 47% relative to sham controls. Repetitive mTBI coupled with hyperthermia at this bregma level decreased lCMR_Glc_ in the primary and secondary motor cortices 48% and 43% respectively. We did not observe significant decreases in glucose utilization between normothermic and hyperthermic repetitive injury groups although values maintained a greater degree of reduction with hyperthermia exposure.

Quantitative analysis of 2DG values confirmed significantly decreased glucose utilization in both repetitive mTBI groups relative to sham, though no significance was attained when comparing the two triple-hit groups. However, there was a marked difference in number of affected ROIs as well as in degree of significance when TBIs occurred under hyperthermic conditions. Nine ROIs at eight bregma levels had significantly decreased lCMR_Glc_ values bilaterally: cingulate cortex (+0.2, −0.8, −1.8), primary and secondary motor cortices (+0.2, −0.8, −1.8, −2.8), retrosplenial cortex (−2.8, −3.8, −4.8, −5.8, −7.3), visual cortex (−4.8, −5.8, −7.3), parietal association cortex (−3.8), and cortical strip (+0.2, −1.8, −2.8, −3.8, −4.8, −5.8, −7.3), which is comprised of the temporal association cortex, and ectorhinal and perirhinal cortices. Subcortically, there were significant glucose metabolic deficits in the caudate putamen (+0.2, −1.8) and striatum (−3.8, −4.8, −7.3). However, significant injury-induced depression in glucose metabolism was not observed in the hippocampus relative to sham controls.

## 3. Discussion

By using the Marmarou model of concussive injury and 2DG autoradiography, we found that three mild concussions over a 7 day span were associated with significantly depressed glucose metabolic activity relative to noninjured controls in several cortical and subcortical regions 8 days post initial TBI. Elevated core temperature at the time of traumatic insult resulted in a greater and more significant degree of metabolic depression relative to control animals. Abnormal hypometabolic alterations in glucose utilization were observed within several diverse, both functionally and spatially, regions. Deficient glucose metabolism occurred in primary and secondary motor, cingulate, retrosplenial, parietal association, and visual cortices, which are structures located medially beneath the sagittal suture and parietal bones (where weight drop impact occurs). These affected areas are responsible for higher-order cognitive processes, such as integrated motor control and goal-directed behavior, reward, spatial decision-making, and navigation. Depression in glucose metabolism was also observed in the caudate putamen and striatum, two subcortical structures that contribute to movement initiation, regulation, and certain types of learning. As we looked more posteriorly, we detected deficient glucose utilization in the medial temporal lobes, specifically within the ectorhinal and perirhinal cortices, areas involved in recognition memory, perception, fear conditioning, and spatial awareness. It would be important to do relevant behavioral tests assessing functional outcomes to determine correlation, if any, between affected structures and resulting behavior.

In the present study, lCMR_Glc_ values appeared somewhat elevated in the sham group (*n* = 7) with the ROI heat maps corroborating this observation. In generating mild concussive TBI, all animals are fasted prior to any surgical procedures to maintain consistent baseline glucose levels, which can alter post-injury outcomes [31]. For this autoradiographic repetitive injury study, all animals are fasted 4 times (3× prior to each TBI or sham surgery, and 1× before 2DG procedure) over a span of 8 days, which may have resulted in hypoglycemia [32]. Furthermore, isoflurane anesthesia has been shown to reduce baseline glucose metabolism [33]. Previous studies have shown that under hypoglycemic conditions, there is an upregulation of glucose transporter-3 (GLUT3) as a cerebral adaptation [34]. Thus, when assessing glucose metabolism during the 2DG procedure (Day 8) greater cerebral expression of GLUT3 may have resulted in a larger degree of glucose uptake. However, given that all randomized experimental groups underwent identical conditions, this potential variability would have been consistent between all animal groups.

There is a great need for establishing evidence-based guidelines to determine when after sustaining mTBI it is safe to return to normal activity. Determination of the rate of glycolysis after injury has been suggested to be a valuable diagnostic tool for patients with TBI [10,35]. The temporal changes of injury-induced depression of glucose utilization represent a period in which the brain is particularly susceptible to further damage and the severity of glucose depression is associated with functional deficits and outcomes [4,15]. Several preclinical and clinical studies have shown worsened outcomes with repeat concussions [10,12,15]. However, the degree to which elevated temperature contributes to glucose metabolic deficits is less known. [^18^F]fluorodeoxyglucose-positron emission tomography (FDG-PET) can be utilized to assess the real-time temporal patterns of glucose metabolism and correlation of functional and metabolic recovery [36]. Quantifying the degree of lCMR_Glc_ may serve as an important biomarker to determine severity of injury, temporal course of greater vulnerability, as well as determination of treatment paradigms.

The effects of small elevations in brain temperature on cerebral metabolism have been previously documented [37,38,39]. In one study, Mickley and colleagues (1997) reported that an induced rise of 2 °C induced by hot/moist air caused a general increase in brain glucose metabolic rate [37]. In a human study, an acute exposure of whole body heat increased glucose and insulin levels in healthy individuals [38]. Regional changes in cerebral metabolism during systemic hyperthermia induced by a liquid-conditioned suit were also reported to increase in several brain regions (hypothalamus, thalamus, corpus callosum, cingulate gyrus and cerebellum) while decreasing in others (caudate, putamen, insula and posterior cingulum) using positron emission tomography [39]. Taken together, these studies emphasize the complex effects of elevated temperatures on regional patterns of glucose metabolism that could impact the long term consequences of TBI especially when present at the time of the insult.

In reference to potential therapeutic interventions, targeted temperature management and therapeutic hypothermia after moderate and severe TBI have been evaluated in multiple randomized controlled trials (RCTs) with inconsistent results [40,41]. In contrast, preclinical translational findings have been encouraging and have provided evidence that cooling the brain after TBI reduces secondary injury mechanisms that contribute to histopathological abnormalities and functional deficits [42]. Currently, there are limited data regarding the effects of brain cooling when core temperature at the time of a mild TBI is elevated. Interestingly, Walter and colleagues [43] have recently reported that selective brain cooling in student athletes in the acute post-concussive period restored an injury-induced reduction of cerebral blood flow (CBF). In that study, post-concussive brain cooling also resulted in the temporary relief of self-reported physical symptoms [43]. Our laboratory has reported in rodents that posttraumatic cooling reduces the cognitive deficits observed after hyperthermic mTBI [24]. Hyperthermia significantly aggravates TBI-induced deleterious events, including a prolonged and a greater degree of depression of glucose metabolism. Thus, targeted temperature strategies that address the potentially deleterious consequences of elevated brain temperatures may be important in the acute management of mild TBI and provide a clinically relevant approach to improving outcomes in this growing patient population.

## 4. Methods

### 4.1. Weight Drop Injury

All studies conducted were approved by the University of Miami Animal Care and Use Committee and by the NIH “*Guide for the Care and Use of Laboratory Animals*”. In total, 23 male Sprague–Dawley rats were designated to one of three groups: Sham + normothermic (*n* = 3), Sham + hyperthermic (*n* = 4), repetitive mTBI + normothermia (*n* = 8), and repetitive mTBI + hyperthermia (*n* = 8). Animals were housed in their home cages for one week before any experimental procedures were conducted. Rats had access to food and water *ad libitum* and were exposed to a 12 h light/dark cycle.

To generate a mild concussive injury, we utilized the Marmarou weight drop model [44,45]. The injury device consists of a segmented brass weight free-falling through a Plexiglas tube. Fasted animals were anesthetized with 3% isoflurane in 30% O_2_/70% N_2_O and placed in a stereotaxic frame. The animal’s head was shaved, and a midline incision was made allowing access to the periosteum covering the vertex of the skull. Once the periosteum was reflected, a stainless-steel disk (10 mm in diameter; 3 mm thick) was secured in the center of the intact skull at the cross-section of bregma and midline with cyanoacrylic adhesive. This disk served as an injury helmet, which protected the animal from skull fracture. After the disk was fully adhered to skull, the animal was removed from the stereotaxic table and maintained under anesthesia via nosecone. Rectal and temporalis muscle thermometers measured and maintained body and brain temperatures using self-adjusting feedback warming lamps. Animals were maintained at normothermic (36.7–37 °C) or hyperthermic (38.7–39 °C) conditions for 15 min prior to injury and for 1 h post injury. Temperature was manipulated using the self-adjusting feedback warming lamps. Desired temperature (37 or 39 °C) was set and warming lamps remained on until the set temperature was reached, at which point the lamps would automatically shut off. If temperature began to drop, the feedback lamps would turn on again. Body and head temperatures were measured and manipulated separately to ensure greater accuracy.

To induce mTBI, animals were removed from anesthesia and placed in the prone position on a foam pad underlying the weight drop device. The injury helmet affixed to the skull was centered directly below the Plexiglas tube where a 450 g weight was allowed to free fall from a height of 1.0 m to generate a mild concussive-like TBI. This height and weight combination results in a brain acceleration of 900 G and a brain compression gradient of 0.28 mm, generating a mild diffuse brain injury. Immediately after injury, the animal was returned to anesthesia and monitored for respiratory changes. Once respiration was normalized, the stainless steel disk was removed and the skull was inspected for fracture. If skull fracture was observed, the animal was discarded from the study. Body and brain temperatures were maintained at 37 or 39 °C for 1 h, at which point anesthesia was terminated. Once conscious, animals were administered a subcutaneous injection of 0.03 mg/kg buprenorphine for pain management and returned to their home cage. Normothermia and hyperthermia injury groups received three injuries: on Day 0, Day 3, and Day 7. The injury interval paradigm selected is clinically representative of repetitive concussion in sports-related injuries. In a prospective study of 16,624 high school and collegiate athletes, McCrea et al. [46] showed that most second or third repeat concussions (79.2%) occurred within 10 days of the initial injury.

### 4.2. ^14^C-2-Deoxy-d-Glucose (2DG) Surgical Methods

Eight days after the first mTBI, fasted animals were reanesthetized for insertion of femoral arterial and venous catheters, as well as a rectal thermometer to maintain normothermic temperatures throughout the duration of the 2DG experiment. Animals were wrapped in a loosely-fitting plaster body cast (Gypsona plaster of Paris), which was secured to a lead block. Anesthesia was terminated and animals were allowed 1 h to recover in a quiet and dark room.

Before the start of the experiment, a blank blood sample was obtained from the femoral artery for baseline radiation levels and baseline glucose concentration. At start, a pulse injection of 20 µCi of ^14^C-labeled deoxy-d-glucose (Perkin Elmer #NEC495) dissolved in 0.2 cc isotonic saline was administered through the femoral vein. Twenty-four arterial blood samples were withdrawn at various intervals over a 45 min period as previously described [47]. After 45 min had elapsed, animals were euthanized via venous injection of Euthasol. Plasma aliquots from samples were assayed for 2DG using a liquid scintillation counter (Beckman) and glucose concentration was assessed via an automated glucose analyzer (YSI 2300). After euthanization, animals were decapitated. Whole brains were quickly removed and frozen over liquid nitrogen vapor. Frozen brains were cryosectioned subserially into 30 µm thick sections (Leica CM1900). A total of 18 bregma levels were collected ranging from 1.2 to −7.3 mm relative to bregma. Brain sections and [^14^C] methylmethacrylate standards were exposed to BioMax MR high-resolution film for 10 days.

### 4.3. Image Processing

Autoradiographic films were digitized at 10 bit intensity precision by a charge-coupled device camera. The camera was interfaced to an advanced image acquisition/analysis system where images were captured at 70 µm/pixel in spatial resolution. [^14^C] methylmethacrylate standards placed on the film were digitized in parallel to permit conversion of optimal density values to activity units of nano-curies per gram of equivalent tissue mass [48]. Images for each experimental group were then transferred to a Linux operating system computer for image registration and averaging of corresponding coronal sections from individual animals.

### 4.4. Region of Interest Analysis and Statistical Analysis

**Figure 4:**
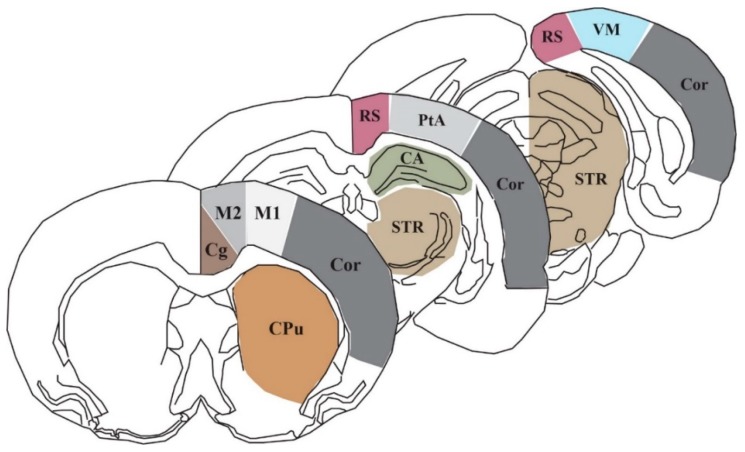
Schematic of three representative coronal sections (+0.7, −3.8, −7.3 mm relative to bregma) showing the 10 autoradiographic regions of interest: CA: cornu ammonis/hippocampus, Cg: cingulate cortex, Cor: cortical strip, CPu: caudate putamen, M1: primary motor cortex, M2: secondary motor cortex, PtA: parietal association cortex, RS: retrosplenial cortex, STR: striatum, and VM: visual cortex.

In order to assess the lCMR_Glc_ of particular regions and structures, we selected nine coronal bregma levels (+0.7, +0.2, −0.8, −1.8, −2.8, −3.8, −4.8, −5.8, −7.3 mm relative to bregma) from the images of each animal that encompass the area of the brain anterior to, posterior to, and directly underneath the impact site where weight drop occurs (approximately 0.0 mm relative to bregma). The Marmarou model of closed head injury utilized in this study generates modest neuronal morphological changes in the supraventricular cortex, which is comprised of several of the ROIs used in this study: cingulate cortex (Cg), primary and secondary motor cortices (M1 and M2, respectively), the retrosplenial cortex (RS), the parietal association cortex (PtA), as well as the visual cortex (VM). We also chose to evaluate lCMR_Glc_ in the caudate putamen (CPu), the striatum (STR), the hippocampus (CA), and cortical strip (Cor). Figure 4 illustrates three representative sections showing the 10 cortical and subcortical ROIs, which contribute to a wide array of functional processes.

Nine digital brain atlas sections were prepared [49]. Sections at the same bregma level from each individual rat were mapped into the brain atlas section at the corresponding bregma level. The image registration procedure was based on an established disparity analysis algorithm whose theory and practical validation were described previously in detail [50,51]. Intergroup differences for lCMR_Glc_ were evaluated using two-way ANOVA. Since the brain sections were mapped into the contours of the corresponding atlas sections, data collection was conducted by superimposing the atlas with the outlined ROI on top of the deformed brain section so that data within the defined ROI could be accurately acquired [52]. We observed no differences between the Sham normothermic and hyperthermic groups. Thus, the groups were combined into a single Sham group for analysis.

## Figures and Tables

**Table 1:** Animal weight and temperature during surgical procedures.

	TBI #1 (Day 0)			TBI #2 (Day 3)			TBI #3 (Day 7)			2DG (Day 8)
	Weight (g)	Head Temp (°C)	Body Temp (°C)	Weight (g)	Head Temp (°C)	Body Temp (°C)	Weight (g)	Head Temp (°C)	Body Temp (°C)	Weight (g)
Sham	335 ± 8	38.09 ± 0.39	38.2 ± 0.35	327 ± 5	38.14 ± 0.42	38.03 ± 0.38	348 ± 8	38.4 ± 0.36	38.3 ± 0.37	344 ± 3
Normothermia	307 ± 3	36.85 ± 0.05	36.68 ± 0.06	304 ± 3	36.88 ± 0.04	36.73 ± 0.07	314 ± 3	36.86 ± 0.04	36.78 ± 0.05	319 ± 4
Hyperthermia	309 ± 2	38.85 ± 0.03	38.79 ± 0.06	310 ± 4	38.84 ± 0.03	38.7 ± 0.05`	313 ± 6	38.85 ± 0.02	38.56 ± 0.28	315 ± 5

**Table 2:** Values represent local cerebral metabolic rate of glucose as mean ± standard error of the mean (µmol/100g/min; *n* = 7–8 per group) of each region of interest (ROI) across nine bregma levels. Two-way ANOVA analyses showed significant differences between sham and triple-hit mild TBI (mTBI) groups. Hyperthermia conferred a higher degree of significance in affected ROIs. * *p* < 0.05, ** *p* < 0.01, *** *p* < 0.001, and **** *p* < 0.0001, where indicated against the sham group. There were no significant differences between the two TBI groups.

Region		Sham	TBI Normo	TBI Hyper
Bregma +0.7 mm				
Caudate putamen	R	115.7 ± 6.6	92.6 ± 14.1	92 ± 12.9
	L	108.1 ± 5.8	90.8 ± 12.6	80.8 ± 7.3
Cortical strip	R	106.3 ± 7.1	92.8 ± 12.6	84.4 ± 7.4
	L	100.6 ± 4.7	83 ± 12.2	74.6 ± 5.1
1° motor cortex	R	100.6 ± 9.4	77.2 ± 11.6	70.6 ± 5.9
	L	95.4 ± 7.2	76.7 ± 11.9	70.2 ± 6.4
2° motor cortex	R	112 ± 9.6	85.1 ± 11.5	88.4 ± 9.5
	L	99.6 ± 7.6	76.8 ± 12	69.4 ± 5.7
Cingulate cortex	R	108.5 ± 7	87.7 ± 10.8	80.8 ± 7.7
	L	98.5 ± 7.4	81.7 ± 11.9	69 ± 6.9
Bregma +0.2 mm				
Caudate putamen	R	117.1 ± 9.2	81.9 ± 12.8 *	74.9 ± 10 *
	L	108.3 ± 8.6	78.7 ± 12.3	65.9 ± 8.4 *
Cortical strip	R	114.1 ± 8.1	86.5 ±12.4	79 ± 8.1 *
	L	106.7 ± 6.5	77.1 ± 11.7	67.5 ± 8.3 *
1° motor cortex	R	112.9 ± 9	77.1 ± 12.8 *	68 ± 7.2 **
	L	99.2 ± 7.3	75.9 ± 12.3	60.9 ± 9 *
2° motor cortex	R	127.2 ± 12.8	81.6 ± 12.7 **	75.4 ± 7.5 **
	L	113.4 ± 5.9	79.4 ± 12.9	63.9 ± 7.4 **
Cingulate cortex	R	133.5 ± 16.3	86.3 ± 11.5 **	77.8 ± 7.9 ***
	L	112.7 ± 11.3	76.1 ± 12.5 *	66.1 ± 5.7 **
Bregma −0.8 mm				
Caudate putamen	R	94.2 ± 7.6	76.2 ± 10.2	63.7 ± 6.5
	L	88.7 ± 8.3	81.1 ± 12	64.8 ± 6.4
Cortical strip	R	102.2 ± 4.6	93.7 ± 12.1	72.6 ± 6.6
	L	104.1 ± 5.6	92.2 ±13.4	73.5 ± 6.8
1° motor cortex	R	118.2 ± 10.6	99.1 ± 9.3	71.4 ± 9.6 **
	L	98.2 ± 6.4	92.5 ± 12.3	67.8 ± 9.1
2° motor cortex	R	132 ± 14.6	110.6 ± 10.8	89.6 ± 10.5 *
	L	103.5 ± 6.2	99.7 ± 13.8	75.7 ± 8.5
Cingulate cortex	R	123 ± 18.1	106.3 ± 8.8	78.5 ± 8.7 **
	L	107 ± 10.3	95.9 ± 14.1	69 ± 6.9 *
Bregma −1.8 mm				
Caudate putamen	R	99 ± 9.2	68 ± 7.4 *	56.9 ±7.5 **
	L	92.5 ± 9.8	70.3 ± 7.5	57.6 ± 5.1 *
Cortical strip	R	110.6 ± 9.9	79.6 ± 7.7 *	71.1 ± 5.5 **
	L	100 ± 7.3	73.4 ± 6	67.2 ± 5 *
1° motor cortex	R	114.9 ± 14	70.9 ± 6.8 **	59.5 ± 5.9 ****
	L	100.2 ± 9.3	63.8 ± 5 **	58.3 ± 2.4 **
2° motor cortex	R	135.3 ± 19.3	94.1 ± 5.2 **	76.7 ± 6.7 ****
	L	121.7 ± 12.2	83 ± 7.2 **	75.8 ± 7 ***
Cingulate cortex	R	127.7 ± 16.1	81.7 ± 5.8 ***	68.1 ± 4.8 ****
	L	116.3 ± 13.9	77.9 ± 7.4 **	61.1 ± 5 ****
Bregma −2.8 mm				
Caudate putamen	R	68.8 ± 8.2	47.6 ± 4.2	42 ± 4.3
	L	71.4 ± 8.3	54.4 ± 6.3	48.8 ± 4.3
Hippocampus	R	74.5 ± 5.4	53 ± 3.8	58.3 ± 4.7
	L	80.9 ± 5.3	58.8 ± 4.8	60.4 ± 4.6
Cortical strip	R	102.9 ± 9.1	74.3 ± 6.8 *	70.4 ± 3.5 *
	L	93.8 ± 7.2	71.3 ± 5.8	67.6 ± 4.3
1° motor cortex	R	104.5 ± 13.5	69.8 ± 8.6 **	56.9 ± 7.6 ***
	L	83.6 ± 9.7	66.2 ± 5	59.4 ± 5.4
2° motor cortex	R	118.2 ± 16.8	84.2 ± 9.2 **	69.8 ± 9 ****
	L	93 ± 12.6	75.9 ± 5.7	68 ± 4.7
Retrosplenial cortex	R	115.8 ± 19.2	80.9 ± 8.3 **	69 ± 6.8 ***
	L	97 ± 13.2	75.1 ± 8.1	60.5 ± 4.2 **
Bregma −3.8 mm				
Striatum	R	137 ± 11.4	91.9 ± 11.8 **	79.2 ± 9.2 ***
	L	129.2 ± 13.1	89.8 ± 12.1 *	77.1 ± 8.6 **
Hippocampus	R	76.7 ± 4.6	57.5 ± 7.1	51.9 ± 6
	L	81.2 ± 6.2	60.9 ± 7.4	53.9 ± 6.8
Cortical strip	R	116.4 ± 11.3	84 ± 12	71.1 ± 8.3 **
	L	112.1 ± 11.1	78.2 ± 11.2	71.6 ± 7.8 *
Parietal association cortex	R	103.9 ± 12.8	77.3 ± 11.7	61.2 ± 8.1 *
	L	93.9 ± 10.7	74.5 ± 10.9	64.9 ± 6.5
Retrosplenial cortex	R	102.3 ± 18.7	80.6 ± 12.7	63.1 ± 7.5 *
	L	96.9 ± 15.4	77.2 ± 13	64.1 ± 6.7
Bregma −4.8 mm				
Striatum	R	110.6 ± 12.1	68.2 ± 8.7 *	64.7 ± 7.5 *
	L	107.5 ± 14.8	67.1 ± 8.6 *	65.6 ± 7.8 *
Hippocampus	R	79.8 ± 9.9	52.5 ± 7.5	51.4 ± 7.1
	L	89.1 ± 11.8	55 ± 6.9	52.1 ± 7.7
Cortical strip	R	129.5 ± 14.8	81 ± 13.4 **	78.2 ± 6.7 **
	L	114.1 ± 12.8	75.5 ± 12.3 *	71.2 ± 6 *
Visual cortex	R	115.1 ± 18.1	73.3 ± 10.9 *	63.6 ± 7 **
	L	120.1 ± 19.6	74.4 ± 10.9 *	67.6 ± 7.4 **
Retrosplenial cortex	R	134.1 ± 21.6	86.4 ± 12.1 **	70.9 ± 8.7 ***
	L	116 ± 15.6	81.1 ± 13.7	69.6 ± 5.4 *
Bregma −5.8 mm				
Striatum	R	83.5 ± 7.6	66.2 ± 8.9	61.5 ± 6.4
	L	84.3 ± 9.1	65.3 ± 8.2	61.3 ± 7
Hippocampus	R	78.9 ± 9.7	56.9 ± 6.9	56.3 ± 6.3
	L	81.3 ± 8.7	57.3 ± 6.3	54.4 ± 6.7
Cortical strip	R	111.9 ± 14.6	78.9 ± 11 *	76.8 ± 8.4 *
	L	103.1 ± 10.7	77.1 ± 11.1	71.4 ± 9.4
Visual cortex	R	95.4 ± 12.3	73.4 ± 10.5	61.4 ± 9.8 *
	L	90.1 ± 11	71.6 ± 9	63 ± 8.8
Retrosplenial cortex	R	102.2 ± 12.7	76.3 ± 10.9	67.6 ± 8.9 *
	L	105.4 ± 9.6	81.2 ± 11.4	66.1 ± 9.1 *
Bregma −7.3 mm				
Striatum	R	83.7 ± 5	67.6 ± 7.8	55.8 ± 7
	L	91 ± 8.2	67 ± 6.7	59.4 ± 7.2 *
Cortical strip	R	118.3 ± 13.6	91 ± 9.5	78.1 ± 5.4 **
	L	117.4 ± 12.2	81.1 ± 10.2 **	76.5 ± 6.6 **
Visual cortex	R	101.2 ± 8.8	88.7 ± 9.8	71.7 ± 6.7 *
	L	111.5 ± 11.6	84.3 ± 9	77.9 ± 5.5 *
Retrosplenial cortex	R	102.6 ± 8.7	84.7 ± 8.2	72 ± 6.8 *
	L	105.2 ± 10.3	83 ± 9.3	73.6 ± 6.1 *

## Abbreviations

TBI	Traumatic brain injury
2DG	^14^C-2-deoxy-d-glucose
mTBI	Mild traumatic brain injury
lCMR_Glc_	Local cerebral metabolic rate of glucose
CBF	Cerebral blood flow
CNS	Central nervous system
ICU	Intensive care unit
ROI	Region of interest
Cg	Cingulate cortex
M1	Primary motor cortex
M2	Secondary motor cortex
RS	Retrosplenial cortex
PtA	Parietal association cortex
VM	Visual cortex
CPu	Caudate putamen
STR	Striatum
CA	Cornu ammonis/hippocampus
COR	Cortical strip
GLUT	Glucose transporter-3
FDG-PET	[^18^F]fluorodeoxyglucose-positron emission tomography
RCTs	Randomized controlled trials
